# Metabolomics of Arterial Stiffness

**DOI:** 10.3390/metabo12050370

**Published:** 2022-04-20

**Authors:** Kaido Paapstel, Jaak Kals

**Affiliations:** 1 Endothelial Research Centre, University of Tartu, 8 Puusepa Street, 51014 Tartu, Estonia; jaak.kals@kliinikum.ee; 2 Department of Cardiology, Institute of Clinical Medicine, University of Tartu, 8 Puusepa Street, 51014 Tartu, Estonia; 3 Heart Clinic, Tartu University Hospital, 8 Puusepa Street, 51014 Tartu, Estonia; 4 Department of Surgery, Institute of Clinical Medicine, University of Tartu, 8 Puusepa Street, 51014 Tartu, Estonia; 5 Surgery Clinic, Tartu University Hospital, 8 Puusepa Street, 51014 Tartu, Estonia; 6 Department of Biochemistry, Institute of Biomedicine and Translational Medicine, Centre of Excellence for Genomics and Translational Medicine, University of Tartu, 19 Ravila Street, 50411 Tartu, Estonia

**Keywords:** arterial stiffness, vascular ageing, pulse wave analysis, pulse wave velocity, metabolomics, metabolism, cardiovascular risk

## Abstract

Arterial stiffness (AS) is one of the earliest detectable signs of structural and functional alterations of the vessel wall and an independent predictor of cardiovascular events and death. The emerging field of metabolomics can be utilized to detect a wide spectrum of intermediates and products of metabolism in body fluids that can be involved in the pathogenesis of AS. Research over the past decade has reinforced this idea by linking AS to circulating acylcarnitines, glycerophospholipids, sphingolipids, and amino acids, among other metabolite species. Some of these metabolites influence AS through traditional cardiovascular risk factors (e.g., high blood pressure, high blood cholesterol, diabetes, smoking), while others seem to act independently through both known and unknown pathophysiological mechanisms. We propose the term ‘arteriometabolomics’ to indicate the research that applies metabolomics methods to study AS. The ‘arteriometabolomics’ approach has the potential to allow more personalized cardiovascular risk stratification, disease monitoring, and treatment selection. One of its major goals is to uncover the causal metabolic pathways of AS. Such pathways could represent valuable treatment targets in vascular ageing.

## 1. Introduction

Blood pressure (BP), traditional blood lipid biomarkers (i.e., total cholesterol, low-density lipoprotein cholesterol, high-density lipoprotein cholesterol, triglycerides), and glucose are widely used in everyday clinical practice for cardiovascular (CV) risk assessment. Unfortunately, the classical biomarkers can only partially explain the distribution of CV risk in the general population. Many metabolic, inflammatory, and hemodynamic drivers of the residual CV risk are thus missed, and a more tailored approach to CV risk stratification is clearly needed. Currently, doctors may be misled by normal cholesterol and glucose blood levels when assessing CV risk in patients whose CV health can also be affected by quite different metabolite species. Prehypertensive patients with increased arterial stiffness (AS) may be unfairly withheld from antihypertensive treatment, and those with excellent vascular function may be overtreated. To overcome these clinical uncertainties, more randomized controlled trials (RCTs) looking beyond traditional CV risk factors are needed.

Recent large-scale RCTs have partially addressed residual inflammatory CV risk by targeting tubuline polymerization [1,2] and neutralizing interleukin-1β [3] and interleukin-6 [4]. Novel NOD-like receptor protein 3 inflammasome inhibitors are being developed with the aim of further limiting the detrimental effects of inflammation [5]. Similar developments are needed to reduce residual metabolic CV risk (risk despite optimal blood glucose and cholesterol control) and residual hemodynamic CV risk (risk despite optimal peripheral BP control). First steps have been taken with the recent SPARTE study, which demonstrated that the AS-driven hypertension treatment strategy can better prevent vascular ageing compared with guideline-based BP control alone [6]. Unfortunately, as the trial lacked sufficient statistical power to demonstrate greater reduction in CV events, it should be replicated with a larger number of patients.

To date, residual metabolic CV risk has been addressed mainly in observational and laboratory research. These studies have often implemented metabolomics, which is an emerging analytical profiling technique [7,8,9]. An increasing number of metabolomics studies link AS to different low-molecular weight metabolite (LMWM) species. The current narrative review aims to provide an overview of existing literature on the relationship between LMWMs and AS. We discuss the current state and future perspectives of this research field. Both targeted and untargeted studies were included. We also propose the novel term ‘arteriometabolomics’ to indicate the research that applies metabolomics methods for studying AS.

## 2. Arterial Stiffness and Its Prognostic Value

AS describes the rigidity of the arterial wall, i.e., the capability of an artery to expand and contract in response to pressure changes. The pressure load of each heartbeat in large conduit vessels is borne mainly by two extracellular matrix proteins: elastin and collagen. An increase in the collagen/elastin ratio during the ageing process is a classical determinant of AS [10]. Other mechanisms that promote AS include low-grade inflammation [11,12], oxidative stress (OxS) [13], endothelial dysfunction (ED) [11], vascular smooth muscle proliferation and stiffness [14], collagen and elastin cross-linking [15], calcification [16], metabolic alterations [17], genetic mutations, and epigenetic abnormalities [18,19]. Thus, both ageing [20,21] and a number of chronic diseases that are associated with the above mechanisms (e.g., hypertension [22], diabetes [23], chronic kidney disease [24]) are closely linked to AS [25].

**Figure 1:**
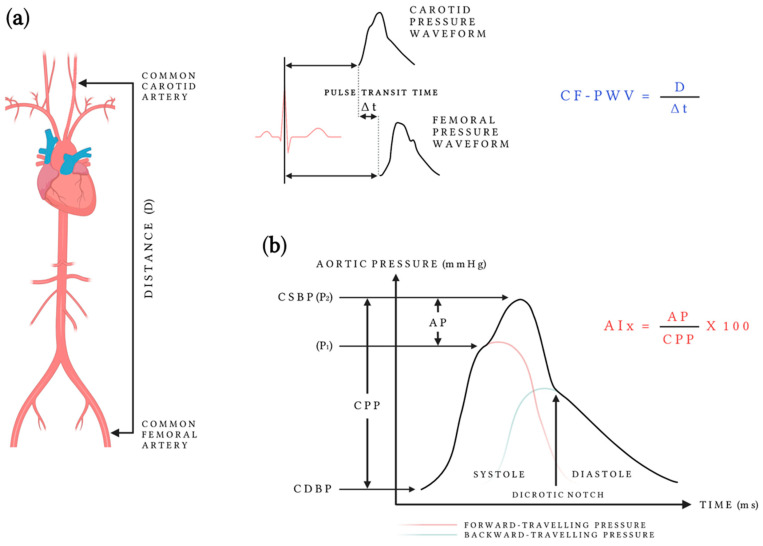
(**a**) Aortic pulse wave velocity measurement with applanation tonometry [28]: pulse transit time is measured from the foot of the carotid pressure waveform to that of the femoral pressure waveform using sequential recordings referenced to the electrocardiogram; the distance between the two recording sites is measured on the body surface and is calculated as the ratio of this distance to the pulse transit time; (**b**) the central aortic waveform is a composite of forward-traveling pressure created by ventricular contraction and backward-traveling pressure reflecting from vascular branch points or sites of impedance mismatch. The phenomenon of left ventricular late-systolic loading can be quantified using Aix—defined as the difference between the second (P2) and first (P1) systolic peaks of the central arterial waveform, expressed as the percentage of CPP [28]. Abbreviations: AIx, augmentation index; AP, augmentation pressure; CDBP, central diastolic blood pressure; cf-PWV, carotid–femoral pulse wave velocity; CPP, central pulse pressure; CSBP, central systolic blood pressure.

AS is an important determinant of target organ damage and an independent CV risk factor. There is strong evidence that aortic stiffness, measured as carotid–femoral pulse wave velocity (cf-PWV), independently predicts CV risk [26,27] and all-cause mortality [28]. Since the waves propagate faster in stiffer vessels than in elastic ones, PWV expresses the time required for an arterial pressure wave to travel between two points in the arterial tree and is one of the simplest and reliable reflectors of AS [29] (Figure 1). Current European guidelines for the management of arterial hypertension state that cf-PWV assessment can help determine asymptomatic organ damage [30]. 

Another reflector of arterial function is the arterial pressure waveform [31] (Figure 1). It is a composite of the forward pressure wave, created by ventricular contraction, and a reflected wave from vascular branch points or sites of impedance mismatch. In elastic arteries, the reflected wave returns to the central aorta in diastole enhancing coronary perfusion pressure [32]. In stiff arteries, however, the reflected wave returns late in the systole, increases central aortic pressure, and impairs coronary artery perfusion [32]. Reduced buffering capacity of the large arteries also leads to excess pulsatility and organ damage in low-resistance vascular beds [33]. From the aortic waveform, various hemodynamic and arterial function parameters can be calculated. Left ventricular late systolic loading, for example, can be quantified using the augmentation index (AIx), which is defined as the difference between the second and first systolic peaks of the central arterial waveform, expressed as the percentage of central pulse pressure. Although cf-PWV is the ‘gold standard’ for measuring AS [29], AIx has also shown independent associations with CV events and all-cause mortality [34].

The methodologies used to determine AS fall under five categories: (1) devices that use a probe or tonometer to record the pulse wave with a transducer, (2) devices using cuffs placed around the limbs or the neck, which record the arrival of the pulse wave oscillometrically, (3) ultrasonography approaches, (4) magnetic-resonance-imaging-based approaches, and (5) invasive measurements with pressure catheters. These methods differ in their applicability, strengths, and limitations, which have been addressed in detail in previous reviews [35,36].

To date, AS assessment has primarily been used for research purposes. Although the independent prognostic value of cf-PWV is now overwhelming, it still remains to be proven whether its measurement truly facilitates clinical decisions. More large-scale trials comparing clinical approaches with and without cf-PWV monitoring are needed. Development of pharmacological agents that directly target AS would also help to pave its way into clinical practice. Current therapeutic options that ameliorate AS include antihypertensives (renin–angiotensin–aldosterone system (RAAS) inhibitors [37], calcium channel blockers [38]), statins [39], and antidiabetic agents [40,41] along with non-pharmacological interventions such as weight loss [42], exercise [42], low salt diet [43], and smoking cessation [44].

## 3. Brief Overview of Metabolomics

Metabolomics is a field of science that combines high-throughput analytical techniques with bioinformatics and focuses on chemical processes involving LMWMs (<1500 Da), including amino acids (AAs), peptides, lipids, carbohydrates, and nucleic and fatty acids (FAs). It reflects changes in the genome, transcriptome, and proteome and thus represents the endpoint of the ‘omics’ cascade [45,46]. The complete collection of LMWMs found in a given organelle, cell, organ, biofluid, or organism is defined as the metabolome. LMWMs in the metabolome are either of endogenous (genetics) or exogenous (nutrition, medication, environment) origin. The endogenous metabolome is highly conserved and represents similar LMWMs, although at different concentrations, across species. The exogenous metabolome, however, is highly variable, reflecting dietary and environmental factors, as well as microbiome composition. Both types of LMWMs can serve as indicators of normal and pathological processes in the organism [45].

Two distinct approaches, targeted and untargeted metabolomics, can be followed to analyze a set of LMWMs in biofluids or tissues. The aim of the targeted approach is to quantify only a preselected set of known LMWMs based on internal or external standards [47]. This approach ensures quantitative accuracy and confidence in LMWM identifications but has a limited reach [47,48]. Untargeted metabolomics, on the other hand, refers to the analysis of all measurable LMWMs in a biological sample but is less accurate in both metabolite identification and quantification [48,49]. There are advantages and disadvantages to both strategies [48], and the choice of approach usually depends on the objectives of the experiment.

To carry out a high-quality metabolomics study, the following steps are usually followed: experimental design, sample collection and extraction, data acquisition, and data analysis and interpretation. Accordingly, (1) a targeted or nontargeted animal or human study is designed; (2) samples of blood, urine, tissue extract, or some other type of biological specimen are collected; (3) mass spectrometry (MS), nuclear magnetic resonance (NMR) spectroscopy, or infrared spectroscopy are employed; and (4) uni-, bi-, or multivariate statistical methods, or a combination of these are used. The two most common analytical platforms for data acquisition and processing in metabolomics are MS and NMR spectroscopy [50]. The NMR approach [51] offers fast LMWM quantification with little sample preparation and very high analytic reproducibility but detects a smaller number of metabolites and requires larger samples compared to MS due to lower sensitivity. The MS approach [52] is often coupled with chromatographic techniques (liquid chromatography, gas chromatography) or with capillary electrophoresis and requires ionization of the analyte before it can be detected. These methods are more time consuming than NRM spectroscopy but are also more sensitive and can identify a wider range of metabolites. Eventually, both types of techniques can provide large-scale and complex datasets on which multivariate statistical analysis (e.g., principal component analysis (PCA), partial least squares regression) and metabolomic pathway analysis are performed to discover hidden patterns and to extract biologically relevant information (e.g., disease-associated metabolic pathways) [53,54,55].

Because of the inherent sensitivity to even subtle changes in the actual phenotype, metabolomics is steadily gaining popularity in different areas of medical research. Altered lipid metabolism in CV disease (CVD), in particular, has been the focus of a number of recent studies [8,56]. Metabolomic profiling seems to improve both the risk stratification and early identification of CVD. It also holds promise for an enhanced understanding of pathophysiological processes (e.g., arterial stiffening) that lead to disorders of the heart and blood vessels.

## 4. ‘Arteriometabolomics’ Approach

**Figure 2:**
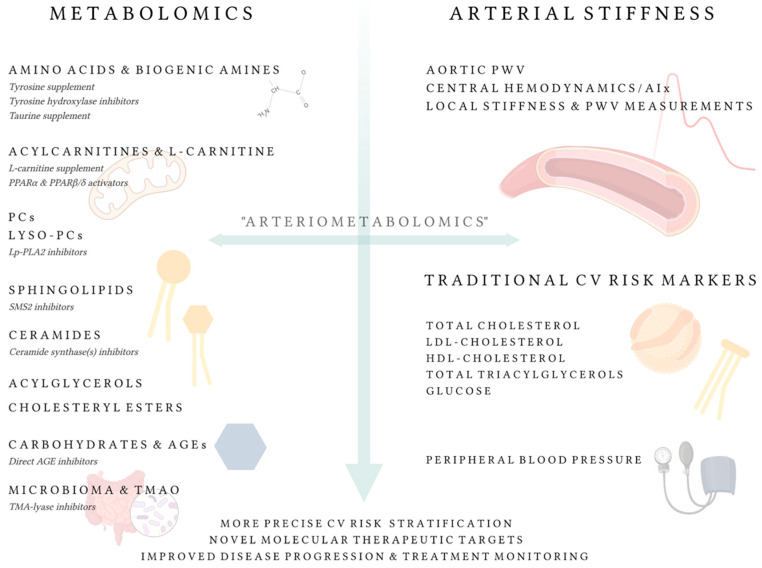
‘Arteriometabolomics’—the crossroad of metabolomics and arterial stiffness research. Metabolites or groups of metabolites that have been associated with arterial stiffness are listed on the left. Metabolism-related compounds that deserve further preclinical/clinical study as potential therapeutics against vascular ageing are displayed in italics. Abbreviations: AGE, advanced glycation end-product; CV, cardiovascular; HDL, high-density lipoprotein; LDL, low-density lipoprotein; Lp-PLA2, lipoprotein-associated phospholipase A2; lyso-PC, lysophosphatidylcholine; PC, phosphatidylcholine; PPAR, peroxisome proliferator-activated receptor; PWV, pulse wave velocity; SMS2, sphingomyelin synthase 2; TMA, trimethylamine; TMAO, trimethylamine N-oxide.

‘Arteriometabolomics’ involves identification and quantification of thousands of LMWMs, metabolic pathways, and their interactions with arterial structural and functional parameters. Current CV risk assessment relies on traditional risk markers (including peripheral BP, blood lipoproteins), but analytical metabolomics methods and AS assessment offer an opportunity to further interrogate the human metabolome and vascular system (Figure 2). The linkage between the arteries and metabolomics can be studied at different levels of the organism—from vascular smooth muscle cell stiffness and metabolism to systemic AS and circulating metabolites. Discoveries made at one level can inspire searches at another. A detailed insight into the roles of individual LMWM species in AS could elucidate the pathophysiology of vascular ageing and lead to novel CV risk markers and potential treatment targets. To date, AS has been associated with numerous LMWMs among which lipids, with an extraordinary diversity in their structure, emerge the most.

### 4.1. Linkage between Low-Molecular Weight Metabolites and Vascular Research: Current State

#### 4.1.1. L-Carnitine, Trimethylamine N-Oxide, and Acylcarnitines

L-carnitine is an endogenous vitamin-like molecule involved in lipid metabolism, synthesized in vivo (from essential AAs lysine and methionine (Met)) and supplemented by diet (e.g., red meat, poultry, dairy) [57]. When carnitine conjugates with long-chain fatty acyl-coenzyme-As (CoAs) on the outer mitochondrial membrane, acylcarnitines (ACs) are formed. Such formation is required in order to transport activated long-chain FAs (>C10) across the inner mitochondrial membrane. Once inside the mitochondrial matrix, AC reconjugates with a CoA molecule after which reformed acyl-CoA undergoes β-oxidation to produce energy. In the case of carnitine deficiency, FA oxidation is impaired [58]. Primary carnitine deficiency is a potentially lethal genetic disorder that causes muscle weakness, myopathy, hypoglycemia, and liver dysfunction [59]. Secondary carnitine deficiency, on the other hand, can result from a decrease in carnitine intake or from an increase in renal excretion and is clinically less pronounced [59].

Subclinical vascular effects of carnitine deficiency and supplementation have been previously studied. Carnitine attenuates free FA-induced ED and modulates platelet activation by limiting the production of endogenous reactive oxygen species (ROS) [60]. Studies in chronic kidney disease patients associate lower circulating levels of carnitine with AS [61,62]. Whether the relationship between carnitine and AS is causal (e.g., through increasing long-chain FA β-oxidation and reducing insulin resistance [63]) is not clear. An ongoing RCT (ClinicalTrials.gov ID: NCT04128969) in adolescents aims to answer this question by measuring the effects of oral carnitine supplementation on cf-PWV, as well as by using the Mendelian randomization technique [64].

Interestingly, L-carnitine supplementation may increase the blood levels of a gut microbe-dependent metabolite trimethylamine N-oxide (TMAO) [65]. Dietary L-carnitine, choline, and betaine have been previously shown to be metabolized by gut microbes to trimethylamine (TMA), which is then carried via the portal circulation to the liver and converted into TMAO [65,66,67]. Several studies indicate that TMAO independently predicts future CVD [68,69] and may promote atherogenesis by affecting cholesterol homeostasis [70] and promoting vascular inflammation [71]. In a recent experimental study by Brunt et al. in mice and humans, TMAO was reported to promote aortic stiffening and age-related ED via superoxide-driven OxS [72]. The authors suggested that TMAO-targeted treatment strategies (e.g., TMA lyase inhibitor 3,3-dimethyl-1-butanol) could have the potential to be effective for vascular ageing [72,73]. In an earlier study in mice by Brunt et al., gut microbiota suppression with antibiotics reversed ED and AS and attenuated vascular OxS and inflammation [74]. Although these findings are intriguing and may potentially shed light on the hidden impact of the gut microbiota on vascular ageing, a considerable number of studies have called causation between TMAO and CVD into question [75,76,77,78]. The microbiome involved in the metabolism of TMAO may play an independent role in disease progression, making TMAO a marker and not an inducer of disease [75]. Further, the amounts of TMA/TMAO or their precursors, which have been frequently used in animal models, exceed the levels present either in a normal diet or in therapeutic supplements [75,79]. The causal role of TMAO in CVD and AS is thus a matter of debate. Besides TMAO, other gut microbiota-derived metabolites (incl. indoxyl sulfate [80,81], *P*-cresyl sulfate [82], phenylacetylglutamine [83], and equol [84]), as well as the composition of the gut microbiota [85,86,87] have been associated with AS.

Some recent metabolomics studies link AS to higher circulating levels of ACs. Accumulation of ACs has been associated with cellular stress [88], inflammation [89], and IR [90] in various study populations. ACs accumulate when the oxidative metabolism of FAs in mitochondria or peroxisomes is impaired (e.g., oxygen deficiency, mitochondrial inborn errors), or when FA release by adipose tissue triglycerides exceeds the capacity of β-oxidation. Short- and odd-chained ACs usually derive from disrupted branched-chain amino acid (BCAA) catabolism. Higher circulating levels of ACs have been associated with increased AS in patients with type 2 diabetes [91], coronary artery disease (CAD) [92], and osteoarthritis (OA) [93], as well as in elderly adults without CVD [94]. PCA-derived factors, primarily composed of medium- and long-chain ACs, were independent determinants of PWV for CAD patients in a targeted metabolomics study by our group [92], as well as for the elderly in a study by Koh et al. [94]. We also found a positive relationship between activity of carnitine–palmitoyltransferase 1 (CPT-1) and cf-PWV for the patients with CAD [92]. The CPT-1 is the key rate-limiting enzyme for β-oxidation of long-chain FAs, and changes in its activity can promote mitochondrial dysfunction and AC accumulation. In a study by Toral et al., up-regulation of CPT-1 (by peroxisome proliferator-activated receptor β/δ (PPAR-β/δ)) prevented free FA-induced ED in isolated vessels and cultured endothelial cells, as well as in mice fed a high fat diet [95]. In a study by Chang et al., however, aortic stiffening was not attenuated by a CPT-1 inhibitor oxfenicine in streptozotocin-induced diabetic rats [96].

#### 4.1.2. Phospholipids

Phospholipids are amphiphilic molecules that serve mainly as structural components in cellular membranes, lipoproteins, and natural surfactants, among others [97]. They consist of FAs and a phosphate-containing headgroup (e.g., choline, ethanolamine, serine (Ser), inositol) attached to an alcohol residue (e.g., glycerol). Plasma FA composition of plasma lipids has been previously associated with AS. Kim et al. showed correlation between FA composition in serum phospholipids and PWV [98]. Among FA composition, linoleic acid (C18:2) was found to be a major independent determinant of AS. In a study by Anderson et al., a PCA-derived component with higher proportions of arachidonic (C20:4), eicosapentaenoic (EPA, C20:5), and docosahexaenoic (DHA, C22:6) and lower proportions of oleic (C18:1), palmitic (C16:0), and linoleic acid (C18:2) levels was linked to lower PWV and systolic BP [99]. The authors concluded that these effects were likely to be mediated either via endothelial metabolism or altered membrane structural properties across the vascular wall. Likewise, lower proportions of serum phospholipid DHA and red blood cell phospholipid EPA were associated with increased AS in studies by Lee et al. [100] and Nishizawa et al. [101], respectively. In a study by Hall et al., an EPA-enriched high-fat meal improved postprandial vascular function, irrespective of changes in OxS [102].

The most abundant phospholipids in cellular membranes and lipoproteins are phosphatidylcholines (PCs) [103]. If a PC molecule becomes partially hydrolyzed by phospholipase A2 or phospholipase A1, one of the two fatty acids bound to the glycerol backbone is removed, and lysophosphatidylcholine (lysoPC) is generated. The production of lysoPC can also result from lecithin-cholesterol acyltransferase (LCAT) activity [104] or hepatic secretion [105]. While some PC and lysoPC species seem to possess pro-atherogenic properties, others may have anti-atherogenic qualities. They also serve as reservoirs and transporters of phosphate, glycerol, and choline. Moreover, both lipid classes participate in cell signaling [106,107] and seem to be associated with vascular ageing.

To date, PCs and lysoPCs are probably the most thoroughly studied phospholipids in regard to AS. In an observational study of patients with symptomatic atherosclerosis, we found that lower serum levels of PC-acyl-acyl C32:2 and several lysoPCs (C16:0, C17:0, C18:0, and C20:4) were related to increased AS, increased resting heart rate, or ED [108]. Kurotani et al. examined the FA composition of serum PC and compared it between Sri Lankan and Japanese patients with diabetes, dyslipidemia, or hypertension. Odd-chain saturated FAs were found to be inversely associated with AS only among the Sri Lankans, possibly due to higher consumption of dairy products and fish in this population [109]. In a study by Petersen et al., however, increases in serum lipid species associated with dairy were positively related to BP and carotid intima-media thickness and unrelated to PWV and AIx [110].

Polonis et al. identified four down-regulated lysoPCs (C16:0, C18:0, C18:1, and C18:2) that were independently associated with high PWV values in patients with hypertension [111]. Positive associations between lysoPCs (C14:0, C16:0, C16:1) and AS, however, have also been reported [91,98,112,113]. The inverse relationship between long-chain lysoPCs and AS is counterintuitive, since the production of lysoPCs depends largely on pro-atherogenic lipoprotein-associated phospholipase A2 (Lp-PLA2). Further, a considerable amount of evidence from ex vivo studies links lysoPCs to ED [114,115,116]. The ability of lysoPCs, present in oxidized low-density lipoprotein (oxLDL) particles, to impair endothelium-dependent relaxation seems to be a function of the chain length of the acyl group [117]. It has been proposed that lower circulating lysoPC levels could reflect increased catabolism and more efficient removal of lysoPCs from blood into tissues [118,119], either in the form of oxLDL or directly from albumin [120]. Moreover, lower activity of LCAT may also partially explain reduced circulating lysoPC levels in atherosclerotic patients [121,122]. The pro- or antiatherogenicity of lysoPC molecule most probably depends on the physical properties of its fatty acid residue (e.g., C16:0, C18:1, C20:4).

#### 4.1.3. Sphingolipids and Ceramides

Sphingolipids and their metabolites are involved in various biological functions and are particularly abundant in tissues of the central nervous system [123]. Like glycerophospholipids, they serve as major plasma membrane structural components (e.g., sphingomyelin (SM)) and as critical signaling molecules (e.g., sphingosine 1-phosphate (S1P), ceramide). De novo sphingolipid synthesis from palmitoyl-CoA and Ser (or alanine, glycine, myristate, stearate) is initiated by Ser palmitoyltransferase. Sequential reactions lead to the generation of ceramides, which are the simplest sphingolipids containing a fatty acid attached to a sphingosine backbone [123,124]. More complex sphingolipids are formed by addition of polar head groups (e.g., phosphocholine, sugar) to ceramide.

Previous studies suggest that sphingolipids may be important mediators of vascular ageing. The sphingolipid precursor Ser was associated with PWV in a large-scale study of female twins by Menni et al. [83]. Ceramides, glycosphingolipids, and S1P have been repeatedly shown to participate in the atherogenic processes [125]. Interestingly, sphingosine-1-phosphate (S1P) and ceramide regulate vascular tone with opposing effects [126]. The cellular balance of these two sphingolipids is called the ceramide/S1P rheostat. A study by Li et al. in rats showed that the imbalance of the aortic ceramide/S1P rheostat may mediate the increase in aortic tone [127]. Ceramides contribute to the NO-mediated arterial dysfunction and elevate ROS production by disrupting the mitochondrial function [128]. A study in mice by Bhat et al. demonstrated that ceramide plays a critical role in phenotype transition and mineral deposition in arterial smooth muscle cells leading to arterial medial calcification and increased PWV [129]. Further, attempts to alleviate vascular dysfunction and atherosclerosis via ceramide synthesis inhibition in animal models have been encouragingly successful [130,131,132].

Glycosphingolipids are formed when sugars (e.g., glucose, galactose) are sequentially added via specific glycosyltransferases (glucosylceramide synthase (GCS), lactosylceramide synthase (LCS)) to ceramide. Like ceramides, this group of lipids can also be causally involved in AS. In a study by Chatterjee et al., inhibition of GCS and LCS drastically and dose-dependently ameliorated atherosclerosis in ApoE^−/−^ mice, and this was accompanied by complete reversal of aortic intima-media thickening and PWV [133]. Subsequently, in a 3-year follow-up study in middle-aged overweight humans, increases in the circulating levels of lactosylceramide, one of the most abundant glycosphingolipids, were independently associated with increases in AS [134]. Circulating lactosylceramide was also an independent predictor of increased AS in subjects with impaired fasting glucose in a study by Jung et al. [135].

SM production from ceramide and PC is catalyzed by SM synthase (SMS). SM concentration is increased in the human atheromatous aorta and coronary vessels [136,137]. In a study by Jiang et al., human plasma SM levels were positively and independently related to CAD, and higher levels were proposed to be a potential marker for atherogenic remnant lipoprotein accumulation [138]. In the Bogalusa Heart Study, a metabolite module related to SM metabolism was associated with PWV in a biracial cohort of 1202 participants [139]. Another large study with 6814 middle-aged asymptomatic adults associated plasma SM levels with quantitative measures of subclinical atherosclerosis, although these associations were lost after adjustment for classical CV risk markers [140]. The authors suggested that plasma SM may partially mediate the associations between traditional risk factors and subclinical atherosclerosis by participating in the intermediate pathways. So far, SMS2 seems to be the most promising SM metabolism-associated therapeutic target with fewer adverse effects than SMS1 isoform in animal models [141]. However, since some SM species (e.g., C23:0, C24:0) have also been associated with a protective effect on CV mortality [142], the true role of SMs in AS still remains to be determined.

#### 4.1.4. Acylglycerols and Cholesteryl Esters

Acylglycerols are esters formed by esterification of glycerol and one to three fatty acids, yielding monoacylglycerol (MAG), diacylglycerol (DAG), or triacyglycerol (TAG). TAGs are the major source of circulating FAs and energy [143]. A positive energy balance (i.e., energy intake exceeding expenditure) leads to an excess of available FAs and TAGs, which may cause IR if accumulating in muscle or liver cells [144]. DAGs are second messengers and intermediates in TAG metabolism and have also been associated with IR [145]. Their role in the development of IR might depend both on the degree of saturation of FA residues in DAG moieties and on the location of the compounds in myocytes [146]. Similarly, a large lipidomics study by Stegemann et al. suggested that associations with higher CV risk are most pronounced for TAGs and cholesteryl esters (CEs) of lower carbon number and double-bond content (i.e., saturated and monounsaturated FAs) [119]. Research addressing associations of molecular composition of acylglycerols with AS, however, is scarce, and more studies applying metabolomics techniques are needed. In the Bogalusa Heart Study, a module composed of MAGs and DAGs, among other glycerolipids, was associated with both AIx and PWV [139]. Almost all of the DAGs in this module consisted of the following acyl groups in different combinations: 16:0, 18:1, 18:2, 20:4. Kulkarni et al. demonstrated that disturbances in the DAG axis independently influence the risk of incident hypertension [147]. The authors suggested that DAG 16:0/22:5 and DAG 16:0/22:6 may be modifiable factors or even targets for treating hypertension. AS, however, was not assessed in that study.

CEs are formed when cholesterol is esterified with long-chain FAs, a reaction catalyzed by acyl-CoA cholesterol acyltransferase (ACAT-1 and ACAT-2) in the cell [148] and LCAT in the blood [149]. CEs serve as the inactive storage or transportation form of cholesterol and are major constituents of blood lipoproteins. Moreover, they accumulate in the atherosclerotic plaques [150], and their circulating levels correlate with development of CAD [151]. Molecular composition of CE species is thus of high interest for CV research. CEs can play a significant role in the pathogenesis of AS, since atherosclerosis is closely linked to arterial stiffening [152]. However, to date, studies investigating associations of individual CE species with AS are lacking.

#### 4.1.5. Amino Acids and Biogenic Amines

AAs are organic compounds that contain a basic amino group, a carboxylic acid functional group, and a side chain that determines the characteristics specific to each AA [153]. They are needed for building proteins and for biosynthesis of compounds such as neurotransmitters and hormones and can also be used as a source of energy. Biogenic amines are formed mainly by decarboxylation of AAs. Prior studies suggest that some AAs and biogenic amines participate in the pathophysiology of AS, and some may even have therapeutic applications.

A metabolomics study in symptomatic peripheral arterial disease patients conducted by our group showed associations between PWV and aromatic AAs (phenylalanine (Phe), tyrosine (Tyr)) [154]. This finding was confirmed in a subsequent larger-scale cohort study of 461 individuals where aromatic AAs, BCAAs (leucine, isoleucine, valine), and histidine (His) were independently associated with PWV [155]. Further, BCAAs and His were also associated with PWV in the large-scale Bogalusa Heart Study [139] as well as in a smaller study by Koh et al. [94]. BCAAs were also correlated with AS in a middle-aged Chinese population [156]. Findings from these studies suggest that the metabolic pathways of BCAAs and aromatic AAs could be involved in AS. Mechanisms underlying the role of BCAAs in AS may involve promotion of inflammation and OxS in endothelial cells [157], insulin signaling disruption, and chronic mTOR (mammalian target of rapamycin) activation that can lead to diabetes [158,159,160], which is a powerful determinant of vascular ageing [23]. Similarly, alterations in glucose metabolism could also at least partially explain the relationship between Tyr and PWV. Tyr is derived from dietary sources as well as from Phe and is a precursor for catecholamines. Circulating Tyr is associated with insulin resistance [161] and can affect BCAA levels, since they compete for the same AA transporter for cellular uptake [162]. Recently, inhibition of Tyr hydroxylase, a rate-limiting enzyme of catecholamine biosynthesis, preserved elastin integrity, attenuated vascular OxS, and reduced the inflammatory infiltrate in mice [163]. Thus, this enzyme could serve as a potential therapeutic target for AS.

Some derivatives of the aromatic AA tryptophan (Trp) and His have also been shown to correlate with AS. Higher circulating levels of indoxyl sulfate, an uremic toxin that is produced by the metabolism of dietary Trp, showed positive associations with aortic stiffness in patients with CAD [164] and type 2 diabetes [165]. Sumatriptan supplementation acutely increased AS in healthy subjects in a small RCT by Vanmolkot and de Hoon, and kynurenic acid was independently associated with AS in patients with atrial fibrillation [166]. His derivatives, however, have shown inverse relationships with vascular function. Urinary 1-methylhistidine, together with β-alanine and L-proline, were independent adverse contributors to AS in young black boys [167], and lower serum levels of 3-methylhistidine were associated with increased aortic stiffness in maintenance hemodialysis patients [168]. Interestingly, a recent study on the influence of tart cherries (Prunus Cerasus) on vascular function found changes in urinary metabolites related to Trp and His metabolism, but there occurred no correlation with AS [169].

Another potential target to attenuate vascular ageing is the arginine (Arg) metabolic pathway. Being the substrate for nitric oxide synthase (NOS), Arg has important nitric oxide (NO) dependent vasodilatory and antithrombotic effects but also several NO independent effects that help to maintain and improve vascular health [170]. For these reasons, it has become a popular dietary supplement, although the CV benefits from long-term supplementation remain questionable. When L-Arg was added to standard postinfarction therapies, it did not improve AS measurements or the ejection fraction and was found to be potentially associated with higher mortality in patients following a first ST-segment elevation myocardial infarction [171]. Asymmetric dimethylarginine (ADMA) is an endogenous inhibitor of NOS isoforms [172] and is released following the proteolysis of Arg-methylated proteins [173]. Higher ADMA levels predispose to ED and are associated with AS [174,175] and carotid intima-media thickness increase [153]. Its isoform, symmetric dimethylarginine (an inhibitor of cellular L-Arg uptake), Arg-to-ADMA ratio, and citrulline-to-Arg ratio also seem to correlate with AS indicators [176,177,178,179].

Homocysteine (Hcy) is an intermediate product in the biosynthesis of the AAs Met and cysteine and a key determinant of the methylation cycle. Higher circulating Hcy levels are associated with AS [180,181], although the independence of this relationship has also been questioned [182]. Hcy may increase AS via direct effects (smooth muscle proliferation, ED, collagen synthesis, and elastolysis) on the arterial wall [183,184,185]. The results from interventional studies with Met loading [186,187,188] and folic acid supplementation [189,190], however, have been ambiguous. Moreover, the effects of folic acid on AS seem to be independent of Hcy levels.

Taurine (Tau) is a biogenic amine with antioxidant and vasodilatory effects [191]. Supplementation of this biogenic amine reduced vascular wall tone, slowed PWV, and led to a decline in AIx and central BP in healthy students [192]. In a study by Ra et al., Tau attenuated delayed post-exercise increase in PWV in young healthy men probably via its antioxidant effects and reversed AIx and ED in another small study in young male diabetics [193]. More recently, the effects of Tau supplementation on BP and on the vascular function were studied in prehypertensive individuals in a placebo-controlled RCT [194]. Tau supplementation significantly decreased BP and improved endothelium-dependent and endothelium-independent vasodilation in these subjects. The hypotensive effect involved the hydrogen sulfide-mediated inhibition of calcium influx.

#### 4.1.6. Carbohydrates and Advanced Glycation End-Products

Glucose (Glc) is a monosaccharide that is vital for fueling both aerobic and anaerobic cellular respiration. Dietary Glc comes in different forms such as mono- (e.g., galactose, fructose, lactose, sucrose), di- (e.g., sucrose, lactose), or polysaccharides (e.g., starch). Excess Glc in the organism is stored as glycogen or converted to fat. These sources help to meet the energy needs during exercise, between meals, and while sleeping. In starvation, Glc can also be derived from the process of gluconeogenesis.

A rapid elevation in blood Glc levels after a meal is an independent risk factor for CVD and a greater risk factor than fasting glucose [195]. Previous studies suggest that AS is increased during both acute and chronic hyperglycemia. The postprandial increase in AS seems to be at least partially dependent on the type (high vs. low glycemic index foods) [196] and amount [197] of consumed carbohydrate. The well-established association between chronic hyperglycemia and increased AS is significantly mediated by the formation of advanced glycation end-products (AGEs) [15]. Glycation is a spontaneous non-enzymatic reaction of reducing sugars with proteins, DNA, and lipids, which forms so-called Amadori products. The Amadori products undergo a variety of irreversible dehydration and rearrangement reactions that lead to the formation of AGEs [15], many of which can be identified and quantified using analytical metabolomics methods. In the arterial wall, glycation contributes to collagen and elastin cross-linking, inflammation [198], and ED [199], which leads to extracellular matrix remodeling. AGEs that have been associated with AS include glucosepane [200], carboxymethyl-lysine [201], pentosidine [202], and methylglyoxal [203]. Some traditional medications such as RAAS inhibitors [204] and statins [205] are known to reduce AGE formation as a by-product of their action [206]. Development of destiffening medicines, which could directly prevent AGE cross-linking or break existing cross-links in the arterial wall, is an area of active research and has a great clinical potential. Currently investigated direct AGE inhibitors include pyridoxamine and epalrestat [207].

#### 4.1.7. Other Metabolites

Uric acid (UA) is a purine derivative and a well established CVD risk marker [208,209]. However, it is not clear whether UA is merely a marker or mediator of CVD and vascular dysfunction [209,210]. Serum UA was recently longitudinally associated with AS in men but not in women [211]. The results from cross-sectional studies have also been equivocal [212,213,214,215,216]. It has been speculated that the association between UA and AS may appear only at high UA levels and may be mediated by xanthine oxidase-induced OxS [211,217].

Polyphenols are naturally occurring compounds found largely in fruits, vegetables, cereals, and beverages [218]. Numerous polyphenols including resveratrol, isoflavones, anthocyanins, and flavan-3-ols, have been shown to be associated with AS [219,220]. Polyphenols can also impact the composition of the gut microbiota and may be metabolized by gut bacteria into bioactive compounds that produce effects on vascular function [221].

Urinary caffeine, paraxanthine, and theophylline excretions were associated with decreased PWV in a study by Ponte et al., suggesting a protective effect of caffeine intake beyond its BP-lowering effect [222]. The acute and long-term effects of caffeine intake on AS and BP, however, are still to be fully elucidated [223].

Urine isocitrate (a citric acid cycle intermediate), hydroxymethylglutarate (a precursor to cholesterol and coenzyme Q10 synthesis), and formiminoglutamate (an intermediate in the catabolism of L-His to L-glutamic acid) were independently associated with AS in Korean adults [224]. The results from coenzyme Q10 supplementation trials have been controversial [225,226,227].

### 4.2. Future Outlook

It is clear from the literature review given above that ‘arteriometabolomics’ is a powerful research tool that allows simultaneous exploration of metabolic and hemodynamic alterations. Combining the increasingly growing knowledge from metabolome analysis with the constantly evolving understanding of AS could lead to the development and validation of novel biomarker panels with a predictive power beyond conventional circulating CVD markers and peripheral BP measurement. In clinical practice, a more detailed overview of subclinical metabolic and hemodynamic (e.g., increased cf-PWV) derangements could be most helpful for low to medium CV risk patients in whom traditional biomarkers/parameters give borderline or equivocal results. It could also help monitor disease (e.g., hypertension, diabetes) progression and treatment efficacy.

Metabolic pathways that prove to be causally associated with AS could emerge as novel therapeutic targets for vascular ageing. Destiffening medicines capable of ameliorating the intrinsic elastic properties of the arterial wall, regardless of BP and cardiac output, would further help protect against CV complications (i.e., reduce residual hemodynamic risk). Recently, the concept of supernormal vascular ageing (SUPERNOVA) was proposed [228]. According to this concept, SUPERNOVA can be diagnosed in individuals who present an exceptionally low AS for their age and sex. The arteries of such subjects are of great scientific interest because determining the unique molecular characteristics of their walls could lead to novel molecular targets. While some progress has already been made in determining therapeutic targets for AS, metabolomics research could further contribute to this endeavor. As reviewed above, the current more intriguing candidate metabolic modulators to improve AS include L-carnitine [64] and Tau [195] supplements, TMA-lyase inhibitors [72], PPARβ/δ activators [95], Tyr hydroxylase inhibitors [163], ceramide synthase inhibitors [141], SMS2 inhibitors [141], and direct AGE inhibitors [207].

A thorough understanding of pathophysiology is a prerequisite for the development of novel therapies for any disease. Metabolomics studies in humans have so far largely focused on systemic shifts in metabolism. Significantly less is known about the local features of metabolism in different organs (e.g., heart, kidneys) and regions of the body (e.g., lower extremity). The research approach based on arteriovenous gradients (AVGs) assessment isolates local changes in the levels of LMWMs of interest from changes in the systemic circulation and is therefore a more direct reflection of the (patho)physiology of the study region. In such an approach, for any given LMWM, venous/arterial ratio <1 is consistent with net uptake by the organ or region of the body, whereas the ratio >1 suggests net release. AVG assessment has been previously used to study local metabolism in the kidneys of patients with chronic kidney disease [229,230] and in the hearts of patients who underwent diagnostic coronary angiography [231]. A study by Lavi et al. associated coronary AVGs of total lysoPC with the coronary endothelial function, setting an example for future local/regional ‘arteriometabolomics’ research [232]. In addition to the heart and kidneys, the metabolism and arterial function (e.g., arterial distensibility, pulse wave imaging, elastography [36]) of other organs and body regions could be similarly studied (e.g., ischemic limb in lower extremity arterial disease patients).

## 5. Conclusions

There is already a significant amount of scientific evidence to support the link between AS and metabolism. Some LMWMs seem to influence the arterial wall through traditional CV risk factors, while others may act independently through both known and unknown pathophysiological mechanisms. The integration of metabolomics methods into arterial wall research broadens our understanding of the pathophysiology of AS and may lead to the discovery of more accurate CV biomarkers and novel destiffening therapies.

## Data Availability

Data sharing not applicable. No new data were created or analyzed in this study.
